# Hsp70.1 carbonylation induces lysosomal cell death for lifestyle-related diseases

**DOI:** 10.3389/fmolb.2022.1063632

**Published:** 2023-02-03

**Authors:** Tetsumori Yamashima, Takuya Seike, Shinji Oikawa, Hatasu Kobayashi, Hidenori Kido, Masahiro Yanagi, Daisuke Yamamiya, Shihui Li, Piyakarn Boontem, Eishiro Mizukoshi

**Affiliations:** ^1^ Department of Psychiatry and Behavioral Science, Kanazawa University Graduate School of Medical Sciences, Kanazawa, Japan; ^2^ Department of Cell Metabolism and Nutrition, Kanazawa University Graduate School of Medical Sciences, Kanazawa, Japan; ^3^ Department of Gastroenterology, Kanazawa University Graduate School of Medical Sciences, Kanazawa, Japan; ^4^ Department of Environmental and Molecular Medicine, Mie University Graduate School of Medicine, Tsu, Japan

**Keywords:** Alzheimer’s disease, calpain-cathepsin hypothesis, hydroxynonenal, non-alcoholic steatohepatitis, type 2 diabetes

## Abstract

Alzheimer’s disease, type 2 diabetes, and non-alcoholic steatohepatitis (NASH) constitute increasingly prevalent disorders. Individuals with type 2 diabetes are well-known to be susceptible to Alzheimer’s disease. Although the pathogenesis of each disorder is multifactorial and the causal relation remains poorly understood, reactive oxygen species (ROS)-induced lipid and protein oxidation conceivably plays a common role. Lipid peroxidation product was recently reported to be a key factor also for non-alcoholic steatohepatitis, because of inducing hepatocyte degeneration/death. Here, we focus on implication of the representative lipid-peroxidation product ‘hydroxynonenal’ for the cell degeneration/death of brain, pancreas, and liver. Since Hsp70.1 has dual roles as a chaperone and lysosomal membrane stabilizer, hydroxynonenal-mediated oxidative injury (carbonylation) of Hsp70.1 was highlighted. After intake of high-fat diets, oxidation of free fatty acids in mitochondria generates ROS which enhance oxidation of ω-6 polyunsaturated fatty acids (PUFA) involved within biomembranes and generate hydroxynonenal. In addition, hydroxynonenal is generated during cooking deep-fried foods with vegetable oils especially containing linoleic acids. These intrinsic and exogenous hydroxynonenal synergically causes an increase in its serum and organ levels to induce Hsp70.1 oxidation. As it is amphiphilic; being water-soluble but displays strong lipophilic characteristics, hydroxynonenal can diffuse within the cells and react with targets like senile and/or atheromatous plaques outside the cells. Hydroxynonenal can deepen and expand lysosomal injuries by facilitating ‘calpain-mediated cleavage of the carbonylated Hsp70.1’. Despite the unique anatomical, physiological, and biochemical characteristics of each organ for its specific disease, there should be a common cascade of the cell degeneration/death which is caused by hydroxynonenal. This review aims to implicate hydroxynonenal-mediated Hsp70.1 carbonylation for lysosomal membrane permeabilization/rupture and the resultant cathepsin leakage for inducing cell degeneration/death. Given the tremendous number of worldwide people suffering various lifestyle-related diseases, it is valuable to consider how ω-6 PUFA-rich vegetable oils is implicated for the organ disorder.

## Hydroxynonenal and cell degeneration/death

Oxidative stress is a complex process. As most of the body’s cellular energy is manufactured in mitochondria by oxidative phosphorylation in the electron transport chain, they are major sites generating reactive oxygen species (ROS). The generation of ROS merely initiates transient oxidative stress. While ROS attack diverse substances, one of the main targets is lipids within biomembranes. ROS attack carbon-carbon double bonds (Ayala et al., 2014) of ω-6 polyunsaturated fatty acids (PUFA) at biomembranes, essentially linoleic and arachidonic acids, and generate 4-hydroxy-2-nonenal (hydroxynonenal). The latter is more stable than ROS which have a relatively short half-life, and can react with targets like senile or atheromatous plaques far from the initial site, because hydroxynonenal is water-soluble but displays strong lipophilic characteristics. So, it has been considered an ultimate mediator of toxic effects, and currently regarded as a secondary and long-lasting oxidative stressor (Uchida, 2003; Bekyarova et al., 2019; Gianazza et al., 2019). Hydroxynonenal is the most intensively studied aldehyde, and may be either protective or damaging to the cells, depending on its concentration (Bekyarova et al., 2019). For example, at low concentrations, it is involved in the control of signal transduction, gene expression, cell proliferation, differentiation, and cell cycle regulation. In contrast, at high concentrations, hydroxynonenal forms adducts with proteins, nucleic acids and membrane lipids, which leads to the long-standing cell disorder and the tissue damage (Humphries et al., 1998; Lashin et al., 2006). As a toxic messenger, it reveals a pathophysiological role that can propagate and amplify oxidative injury and induce cell degeneration/death. The cell damage in certain organ can lead to damage of other organs and cause severe complications. Accordingly, it is reasonable to speculate that individuals with certain lifestyle-related disease have an increased risk for other diseases.

ROS can continuously cause the tissue damage when their product hydroxynonenal overcomes the antioxidant defense system (Pizzino et al., 2017). Glutathione S-transferases, alcohol dehydrogenases, and aldehyde dehydrogenases (ALDH), are representative enzymes which are capable of degrading hydroxynonenal (Pham et al., 2002; Castro et al., 2017; Zhang and Forman, 2017). Hydroxynonenal detoxification by glutathione S-transferases is reduced with the age-dependent enzyme depletion, which in turn may facilitate toxicity of hydroxynonenal (Schaur et al., 2015). The mitochondrial enzyme, ALDH2 is the key enzyme being involved in the detoxification of not only ethanol’s metabolite ‘acetaldehyde’, but also another aldehydic product ‘hydroxynonenal’ (Edenberg, 2007; Joshi et al., 2019).

**FIGURE 1:**
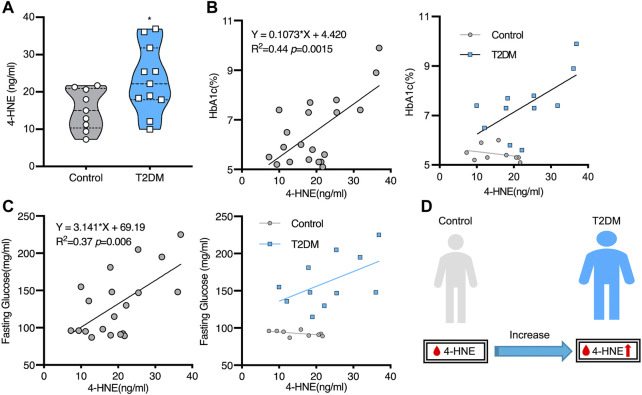
Increased serum hydroxynonenal (4-HNE) level in the patients with type 2 diabetes (T2DM) Panel **(A)** The serum 4-HNE level was significantly higher in T2DM patients, compared to the non-diabetic subjects (Control). Panels **(B)** and **(C)** The simple linear analysis shows that the serum 4-HNE level was positively correlated with HbA1c **(B)** and fasting glucose **(C)**. Panel **(D)** The serum 4-HNE level was closely related to the occurrence of T2DM. Reprinted with permission from Lou et al. (2020).

As millions of East Asians carry Glu504Lys loss of function mutation (ALDH2*2), they are prone to lose ALDH2 activity and accumulate hydroxynonenal (Chen et al., 2015; Joshi et al., 2019). Those with ALDH2*2 mutation cannot clear toxic aldehydes, so mitochondrial dysfunction occurs because of additional ROS generation. Oxidative stress and energy failure synergically cause various diseases. For instance, ALDH2*2 mutation was previously demonstrated to be a risk factor for Alzheimer’s disease (Wang et al., 2008; Chen et al., 2019). Kamino et al. found that subjects with ALDH2*2 are prone to develop late-onset Alzheimer’s disease, by interacting with apolipoprotein E allele 4 (ApoE ε4) (Kamino et al., 2000). Furthermore, serum hydroxynonenal level was significantly higher in type 2 diabetes. Accumulation of hydroxynonenal showed a positive correlation with both increased hemoglobin A1c (HbA1c) and fasting glucose levels in human patients (Figure 1). Accordingly, Lou et al. (2020) suggested that hydroxynonenal is one of the causative factors of type 2 diabetes. In addition, the occurrence of non-alcoholic steatohepatitis (NASH) was reported to be closely related to hydroxynonenal (Bekyarova et al., 2019). Using diverse experimental paradigms, Seike et al. recently found that hydroxynonenal causes hepatocyte death by disrupting lysosomal membrane integrity (Seike et al., 2022). Taken together, the cellular and molecular mechanisms of hydroxynonenal-induced organ injury should be elucidated in detail with regard to the progression of lifestyle-related diseases.

Monkey brains after the long-term injections of hydroxynonenal, showed the widespread neuronal degeneration/death due to the lysosomal membrane permeabilization/rupture without an implication of amyloid *ß*. Therefore, Yamashima. (2021) recently suggested such a concept that the exact causative substance of Alzheimer’s disease might be not amyloid *ß* but hydroxynonenal. Furthermore, the same monkey experimental paradigm indicated that the major pathophysiological mechanism behind the development of Langerhans cell degeneration/death in type 2 diabetes is oxidative stress being mediated by hydroxynonenal (Boontem and Yamashima, 2021). Since glucose-induced insulin secretion is impaired by hydroxynonenal, the resultant hyperglycemia conceivably causes an increase of oxidative stress with the subsequent acceleration of lipid peroxidation which facilitates generation of hydroxynonenal to worsen type 2 diabetes (Miwa et al., 2000). In addition, both experimental and clinical studies have affirmed that hydroxynonenal-modified proteins play crucial roles in the progression of chronic liver diseases (Barrera et al., 2015; Wang et al., 2015). However, the molecular mechanisms of both hepatocyte degeneration/death and progression from simple fatty liver to NASH have remained unclear.

When the symptoms and/or signs of Alzheimer’s disease, type 2 diabetes, and NASH appear, usually the disease has already been present for many years. For example, in Alzheimer’s disease, four stages of the disease progression have been proposed: 1) pre-disease stage without any pathophysiological alterations detectable, 2) pre-clinical stage with amyloid *ß* and hyperphosphorylated Tau (pTau) depositions but without cognitive decline, 3) stage of mild cognitive impairment (MCI), and 4) the dementia stage (Chen and Zhong, 2013). Similar processes of pre-diabetes or simple steatosis should be present in type 2 diabetes and NASH, respectively. It seems worthwhile to discuss whether and how hydroxynonenal is responsible for the development of these lifestyle-related diseases.

Heat shock proteins (Hsp) were accidentally discovered by heat shock in *Drosophila melanogaster* in 1962 by the epoch-making work of Ritossa in Italy (1962). Other than thermal stress, Hsp expression is induced by such insults as ischemia, heavy metals, nutrient deprivation, irradiation, infections, inflammation, and exposure to organics and oxidants (Lindquist and Craig, 1988). The Hsp70 family is evolutionarily the most conserved subfamily, and the major stress-inducible member of this family is Hsp70.1 (also called Hsp70, Hsp72). Hsp70.1 is responsible for folding newly synthesized polypeptides under physiological conditions and misfolded proteins under stress. To carry out these tasks, Hsp70.1 employs a large number of cochaperones and adapter proteins. Stress-induced upregulation of Hsp.1 promotes cell survival against insults that have the potential to induce cell damage. Hsp70.1 plays a key role to maintain intracellular protein homeostasis. It has five activities in the cell: 1) binding misfolded proteins to favor protein refolding cycles and prevent their aggregation (Young et al., 2004), 2) bringing unfolded proteins through membranes to enable delivery of cargo to organelles (Hohfeld and Hartl, 1994), 3) recruiting proteins to the proteasome for turnover (Demand et al., 1998), 4) transporting proteins to the endosome/lysosome for chaperone-mediated autophagy (Majeski and Dice, 2004), and 5) preserving lysosomal membrane integrity (Kirkegaard et al., 2010). Lysosome membrane integrity is protected by Hsp70.1, Lamp-1/2, LIMP2, CD63, etc. Lysosome membrane disintegrity may occur by the degradation of Hsp70.1 or Lamp-1 in response to ROS, proteases such as caspases and calpains, as well as by the cytoskeleton disruption and changes in sphingolipid composition. Lysosomal membrane integrity is affected by both sphingolipid composition and acid sphingomyelinase (EC3.1.4.12) (Gabande-Rodriguez et al., 2014). Acid sphingomyelinase resides inside lysosomal lumen and its hydrolytic activity is stabilized by bis(monoacylglycero)phosphate (BMP) (Linke et al., 2001). The Hsp70.1-BMP interaction enhances association of BMP with acid sphingomyelinase, which can activate this enzyme so that it breaks down sphingomyelin to generate ceramide (Kirkegaard et al., 2010). Ceramide protects the lysosomal membrane from rupturing, because the increased concentration of ceramide possibly facilitates fusion of lysosomes with other intracellular vesicles and cell membranes (Heinrich et al., 2000; Kirkegaard et al., 2010; Yamashima, 2013).

This review aims to indicate hydroxynonenal-induced cell degeneration/death as a common cause of Alzheimer’s disease, type 2 diabetes, and NASH. Here, we discuss such a common cascade as ‘oxidative stress—generation of hydroxynonenal—calpain activation—Hsp70.1 carbonylation—cleavage of Hsp70.1—lysosomal membrane disintegrity—cathepsin release—cell death’ (Oikawa et al., 2009; Yamashima and Oikawa, 2009) which leads to disorders of the brain, pancreas, and liver. The authors are convinced that this is exactly the first review discussing that the above three lifestyle-related diseases may occur by the same culprit, ‘hydroxynonenal’.

## Alzheimer’s disease

Alzheimer’s disease causes severe memory loss and progressive dementia due to widespread loss of neurons and synapses, which was thought to be caused by amyloid plaques, neurofibrillary tangles, and amyloid angiopathy (Citron, 2002). Early-onset, familial Alzheimer’s disease due to the genetic aberrations accounts for less than 5% of the total cases. In contrast, sporadic Alzheimer’s disease of late-onset with aging, accounts for more than 95%. Apolipoprotein E (APOE 19q32.13) ε4 allele has been considered the main genetic disorder responsible for the sporadic form. ALDH2*2 is the most common mutation in ALDH2 gene. Ohsawa et al. (2008) found that ALDH2*2 mutation mice (Aldh2^−/−^) revealed Alzheimer-like molecular changes such as increased hydroxynonenal generation and amyloid *ß* formation, and Aldh2^−/−^ mice were associated with age-dependent neurodegeneration and memory loss. The epidemiological study in China has identified ALDH2*2 as a causative factor for Alzheimer’s disease (Wang et al., 2008). Moreover, a case control study from Japan revealed that ALDH2*2 was associated with the occurrence of late-onset Alzheimer’s disease (Kamino et al., 2000). Recent meta-analysis also demonstrated the positive correlation between ALDH2*2 and occurrence of Alzheimer’s disease (Chen et al., 2019).

**FIGURE 2:**
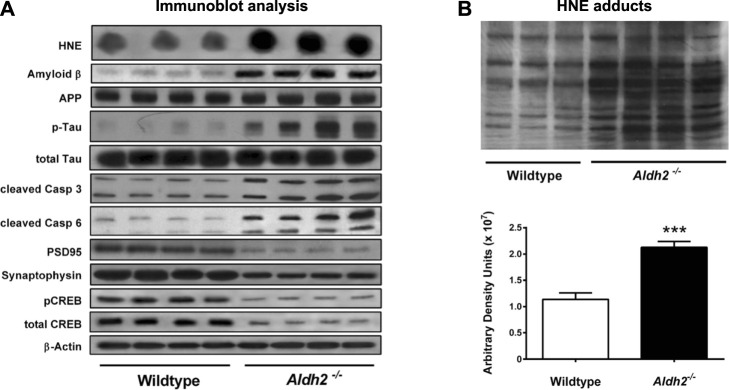
Molecular markers and hydroxynonenal (HNE) adducts in the hippocampus of Aldh2^−/−^ mice. Panel **(A)** Immunoblot analysis of hippocampal homogenates from wildtype or Aldh2^−/−^ mice. Aldh2^−/−^ mice showed increase of Alzheimer’s disease-associated markers (top part) and decrease in synaptic markers (bottom part), compared to the wildtype. Panel **(B)** Aldh2^−/−^ mice showed a significant increase of HNE adducts, compared to the wildtype. APP, amyloid precursor protein; P-tau, hyperphosphorylated tau protein; Casp, caspase; PSD95, postsynaptic density protein 95; CREB, cyclic AMP response element binding protein. Reprinted with permission from D’Souza et al. (2015).

As a hallmark of Alzheimer pathology and a key event in early cognitive decline in the disease progression, both synaptic dysfunction and loss of synapses occur prior to the formation of senile plaques which have been thought to be associated with neuronal death. As an index of neurodegeneration and synaptic loss, D'Souza, et al. (2015) observed decreased levels of both the postsynaptic protein PSD95 and the presynaptic protein synaptophysin in the hippocampus of very young (3 months old) Aldh2^−/−^ mice (Figure 2). The latter showed increased level of hydroxynonenal, concomitant with age-dependent, progressive cognitive decline and hippocampal atrophy. Interestingly, in addition to Alzheimer-like pathological changes, they found significant vascular alterations such as age-dependent increases in hydroxynonenal adducts and monomeric amyloid *ß* in the brains of Aldh2^−/−^ mice (D'Souza et al., 2015). Amyloid *ß* angiopathy is a common pathological feature occurring in 60%–90% of Alzheimer patients (Kalaria and Ballard, 1999; Attems and Jellinger, 2014). The presence of cerebral amyloid angiopathy significantly facilitates cognitive decline in the early Alzheimer’s disease (Esiri et al., 1999). It is likely that both endothelial dysfunction and arterial hypercontractility are associated with chronic hypoxia of the brain.

**FIGURE 3:**
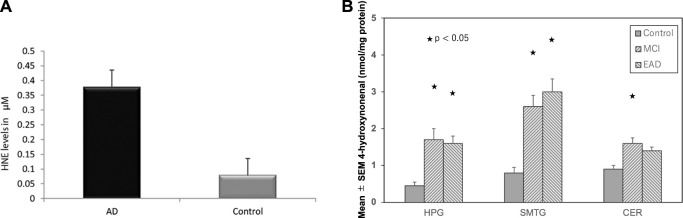
High hydroxynoneal levels in Alzheimer’s disease (AD) and mild cognitive impairment (MCI). Panel **(A)** The plasma hydroxynonenal level (HNE) in the patients with Alzheimer’s disease (AD) and the control subjects. (Cited from Rani et al., 2017). Panel **(B)** Tissue hydroxynonenal concentrations in the hippocampus/parahippocampal gyrus (HPG), superior and middle temporal gyrus (SMTG), and cerebellum (CER) in the patients of MCI, early Alzheimer’s disease (EAD), and age-matched control subjects. Adapted with permission from Williams et al. (2006).

In 2001, McGrath et al. (2001) reported high levels of hydroxynonenal in the Alzheimer’s disease patients (6.0–25.2, median 20.6 μmol/L), compared to the control subjects (3.3–14.5, median 7.8 μmol/L) by the method of Esterbauer and Cheeseman. (1990). In 2017; Rani et al. (2017) confirmed a significant increase in hydroxynonenal level in the plasma of Alzheimer’s disease patients (.38 ± .26 µM), compared to the control group (.08 ± .05 µM) by the method of Benedetti et al. (1980). Two different methodology showed approximately 3∼5 fold increase of the serum hydroxynonenal levels in Alzheimer’s disease, compared to the control (Figure 3A). In addition, tissue hydroxynonenal concentration was significantly higher in the autopsy brain of patients with early Alzheimer’s disease and MCI relative to the healthy subjects (Figure 3B) (Williams et al., 2006). Especially, amyloid *ß* plaques and neurofibrillary tangles in the hippocampus were shown to contain abundant hydroxynonenal (Sayre et al., 1997; Ando et al., 1998).

The oxidative stress hypothesis of Alzheimer’s disease (Butterfield, 1997; Markesbery, 1997; Praticò, 2008) suggests that oxidative damage may be crucial for its occurrence. Hydroxynonenal would be continuously generated as the long-term oxidative stressor. Both free hydroxynonenal and its protein adducts were reported to accumulate in the brains of patients with Alzheimer’s disease (Lovell et al., 1997; Montine et al., 1997; Sayre et al., 1997; Markesbery and Lovell, 1998; McGrath et al., 2001; Fukuda et al., 2009; Reed et al., 2009; Butterfield et al., 2010). Joshi et al. (2019) suggested such a concept that both ALDH2 inactivating mutation and chronic excessive ethanol intake are potential contributors to Alzheimer’s disease progression. After 11 weeks-intake of ethanol, amyloid β42 levels in the brain were higher in ALDH2*2 mice, compared to the control mice. However, even in the absence of ethanol exposure, Aldh2^−/−^ mice showed an increased hydroxynonenal level and developed Alzheimer’s disease-like pathology (D’Souza et al., 2015). Many studies have supported that the generation of hydroxynonenal preceeds to the occurrence of Alzheimer’s disease, although the underlying molecular mechanism was uncovered until recently.

**FIGURE 4:**
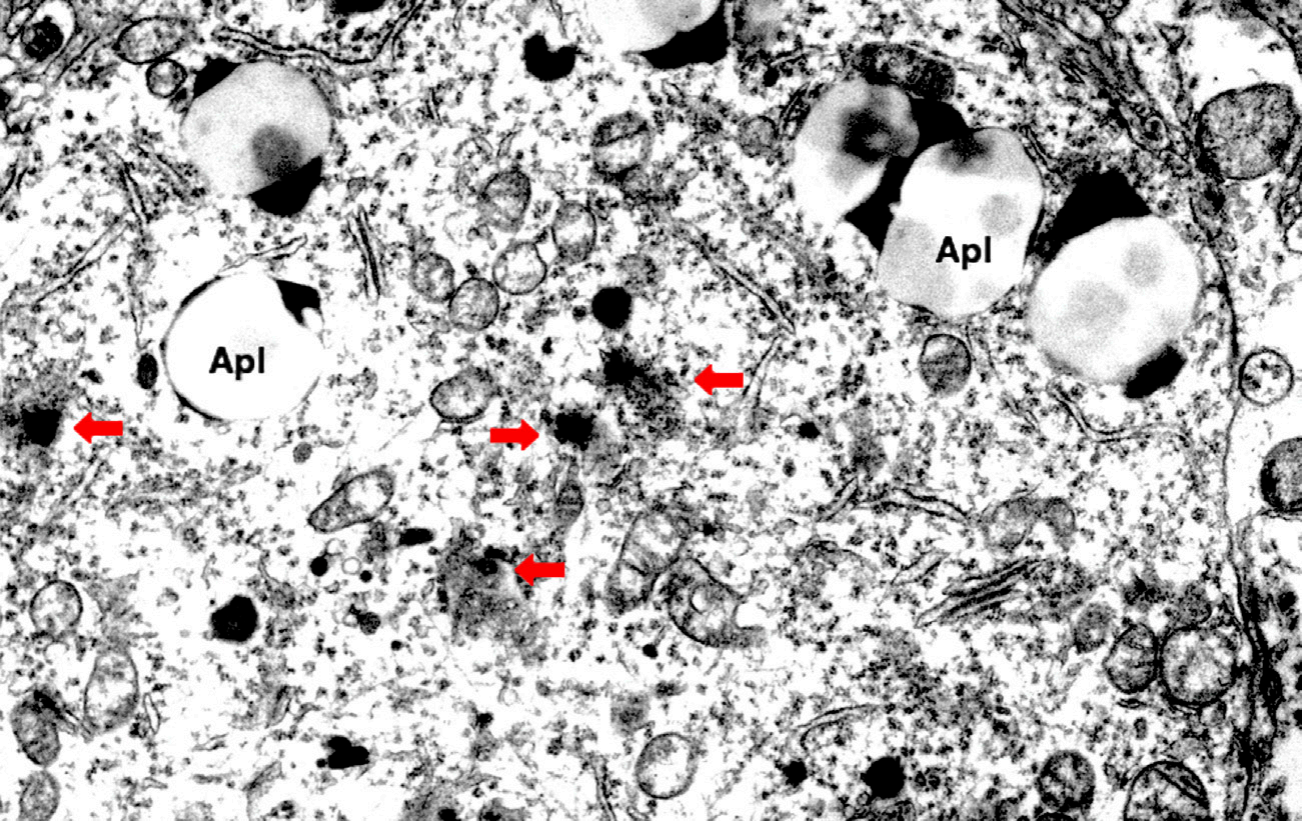
Electron microphotograph of the lysosomal rupture being observed in the cortical neuron of human Alzheimer patient. Red arrows are lysosomal membrane rupture/permeabilization, which shows a remarkable contrast to the intact lysosome (circles). Apl: autophagolysosome (Reprinted from Yamashima (2020)).

The lysosomal membrane destabilization was thought to be responsible for the oxidative stress-induced cell damage, since ROS were well known to induce leakage of the lysosomal content (Zdolsek and Svensson, 1993; Antunes et al., 2001; Dare et al., 2001; Persson et al., 2003). However, implication of ROS for the programmed cell death in diseases was not elucidated in detail until the formulation of the ‘calpain-cathepsin hypothesis’ by the authors (Yamashima et al., 1998; Yamashima, 2000; Oikawa et al., 2009; Yamashima and Oikawa, 2009). Although extremely rare to encounter in the advanced stage of Alzheimer’s disease, Yamashima. (2016) found evidence of lysosomal membrane permeabilization in the cortical neuron of the Alzheimer patient (Figure 4) with the aid of Prof. R.A. Nixon in New York. Furthermore, by the consecutive injections of the synthetic hydroxynonenal to Japanese macaque monkeys, Yamashima (2021) recently observed similar lysosomal disorder and widespread neuronal death as seen in human Alzheimer patients. Accordingly, he suggested such an idea that hydroxynonenal might be a real culprit of Alzheimer’s disease, and amyloid accumulation may appear as a byproduct of lysosomal and autophagy failure which was brought by the calpain-mediated cleavage of the oxidized Hsp70.1. ROS can induce lipid peroxidation of linoleic and arachidonic acids being involved in biomembranes, and generate hydroxynonenal *in vivo*. In addition, hydroxynonenal is generated during deep-frying of the ω-6 PUFA-rich vegetable oils. Accordingly, intake of the excessive deep-fried foods or high-fat diets may lead to an elevation of the hydroxynonenal concentration in both the serum and organ (Thaler et al., 2012; Yamashima et al., 2020).

## Type 2 diabetes

Globally, the epidemics of not only Alzheimer’s disease but also type 2 diabetes are increasing worldwide and have huge costs, human suffering, and economic burden. Perlmuter et al. (1984) reported that memory deficiencies in aging, non-insulin-dependent diabetic patients were associated with higher HbA1c levels. Thereafter, abundant epidemiological and molecular evidence suggests considerable overlap in risk, comorbidity and pathophysiological mechanisms between these two diseases (Biessels et al., 2006a; b). Since Alzheimer’s disease and type 2 diabetes share many pathophysiological features such as insulin resistance, amyloid aggregation, inflammatory stress, and cognitive disturbances, there should be common pathogenic processes. Accordingly, the nickname of ‘type 3 diabetes’ for Alzheimer’s disease has been proposed, but the reason of this close relation has been unknown until now. It still remains unelucidated whether Alzheimer’s disease and type 2 diabetes are parallel disorders due to coincidental events with aging, or synergistically linked by pathogenic vicious circles (Arnold et al., 2018). In the patients with Alzheimer’s disease, increased amyloid *ß* and ROS levels enhance lipid peroxidation, thus increasing the level of toxic hydroxynonenal (Butterfield et al., 2002). Hydroxynonenal levels are significantly high in the autopsy samples of hippocampus which were resected from the patients with MCI and early stages of Alzheimer’s disease (Figure 3B) (Williams et al., 2006). Especially, amyloid plaques and neurofibrillary tangles involved in the *postmortem* hippocampus were shown to contain abundant hydroxyonenal (Sayre et al., 1997; Ando et al., 1998).

Insulin resistance is an essential factor for type 2 diabetes, and is also a common feature of Alzheimer patients with or without type 2 diabetes. So, for understanding the association between Alzheimer’s disease and type 2 diabetes, the phenomenon of insulin resistance is essential. As insulin receptor is widely distributed throughout the brain, insulin plays a crucial role as cerebral safeguard for neuronal physiology and mental health (Berlanga-Acosta et al., 2020). Its role in the brain is not restricted to the control of glucose uptake and utilization for energetic purposes, because insulin has also pro-survival, trophic, and anti-apoptotic effects (Kandimalla et al., 2017). In addition, insulin signalling in the brain regulates metabolic pathways in the liver and adipose tissue, and these effects are thought to be mediated by the action of insulin in the hypothalamus (Arnold et al., 2018).

Either free radicals (intracellular stimuli) or proinflammatory cytokines (extracellular stimuli) activate c-Jun N-terminal kinase (JNK), which facilitates serine phosphorylation in the insulin response substrate protein, IRS-1 (Berlanga-Acosta et al., 2020). In the brain, JNK is activated also by amyloid *ß* and pTau. Since phosphorylation of serine residues inhibits the interaction of IRS-1 with the insulin receptor, the response to insulin would be disturbed. JNK activation promotes the proinflammatory cytokine transcription, which in turn enhances oxidative stress and accumulation of amyloid *ß* and pTau, and ultimately enhances the insulin resistance (Berlanga-Acosta et al., 2020). Insulin resistance in type 2 diabetes has been defined as ‘reduced sensitivity in human body to the action of insulin’ (Goldstein, 2002), while insulin resistance in the brain can be defined as ‘the failure of neurons to respond to insulin’ (Mielke et al., 2005). Accordingly, insulin resistance and impaired cerebral glucose metabolism are a core feature of both type 2 diabetes and Alzheimer’s disease. Insulin is one of the key players of a vicious circle perpetuating type 2 diabetes and Alzheimer’s disease.

Although the causative substance of oxidative-induced insulin resistance long remained unclear, Mattson (2009) first reported implication of hydroxynonenal in the insulin resistance. For example, accumulation of hydroxynonenal-modified proteins occurs in the pancreatic β-cells of GK rats as a result of hyperglycemia (Ihara et al., 1999). Moreover, mice lacking the hydroxynonenal-conjugating enzyme glutathione S-transferase exhibit accumulation of hydroxynonenal in multiple tissues and spontaneously develop obesity and insulin resistance (Singh et al., 2008). Hydroxynonenal can form covalent adducts on IRS-1 and Akt, and activate MAPK-signaling pathways to impair IRS activation (Leonarduzzi et al., 2004; Demozay et al., 2008; Shearn et al., 2011). Accordingly, hydroxynonenal and other lipid peroxidation byproducts impair glucose-stimulated insulin secretion in isolated β-cells (Miwa et al., 2000) and cause β-cell death (Lenzen, 2008). In addition, hydroxynonenal is increased in adipocytes during obesity in which it may impair the function of key proteins involved in lipid metabolism (Grimsrud et al., 2007), and exhibit impaired insulin action (Demozay et al., 2008). As insulin is the main regulator of carbohydrate and fat metabolism, impairment of its function leads to insulin resistance. Pillon et al. (2011) demonstrated that adduction of insulin by hydroxynonenal induce structural and functional changes of human insulin, and this also indicated a putative role of hydroxynonenal in the development of insulin resistance.

**FIGURE 5:**
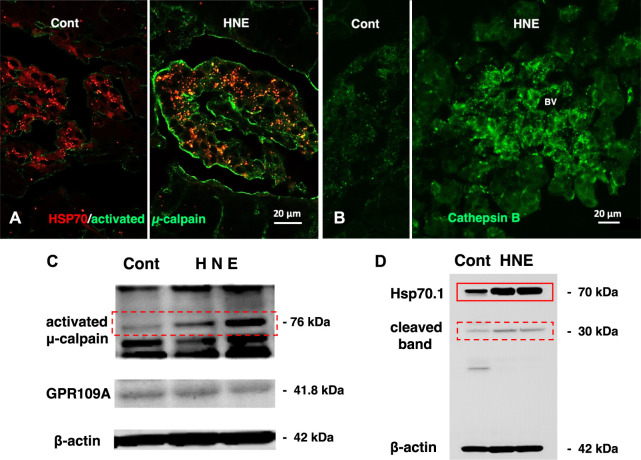
Calpain activation, Hsp70.1 cleavage, and cathepsin B leakage in the monkeys after the consecutive hydroxynonenal (HNE) injections. Panel **(A)** Activated μ-calpain immunoreactivity (green) is negligible before HNE injections (Cont), whereas μ-calpain activation occurred after HNE injections (HNE), being consistent with the Western blotting data (Panel **(C)**, activated μ-calpain). After HNE injections, activated μ-calpain immunoreactivity (green) is colocalized with Hsp70.1 immunoreactivity (red), showing a merged color of yellow (HNE, yellow). Panel **(B)** Cathepsin B is stained as tiny granules in the control Langerhans islet (Cont), whereas stained as coarse granules with the perigranular immunoreactivity after HNE injections (HNE), which indicates lysosomal membrane rupture/permeabilization. Panel **(C)** By Western blotting, μ-calpain is activated after HNE injections (dot rectangle), compared to the control (Cont). As this anti-μ-calpain antibody recognizes only activated form of μ-calpain, but not inactivated form, positive bands indicate activation of μ-calpain. Panel **(D)** In response to HNE injections, not only Hsp70.1 main bands (rectangle) but also cleaved Hsp70.1 bands of 30 kDa (dot rectangle) are increased, compared to the control. Reprinted with permission from Boontem and Yamashima (2021).

Amyloid *ß* and insulin have a close relation; the former metabolism is impacted by the latter and the threshold of insulin receptor sensitivity. In contrast, amyloid *ß* interferes with insulin binding to its receptor (Berlanga-Acosta et al., 2020). Previous studies have linked insulin resistance with cognitive impairment and cerebral atrophy (Burns et al., 2012; Moran et al., 2013). Insulin resistance has been highly correlated with the reduced rate of glucose metabolism in the brain of patients with type 2 diabetes (Baker et al., 2011; Roberts et al., 2014). Further, in the patients with type 2 diabetes who suffer from MCI, a decrease of the cerebral blood flow has been observed (Chau et al., 2020). Both brain and pancreas are particularly susceptible to lipid oxidation as a result of high oxygen consumption in each organ. Although evidence concerning a relationship between the two diseases at the molecular level is still not sufficient, fatty acid peroxidation is linked to either Alzheimer’s disease or type 2 diabetes. Especially, lipid peroxidation product hydroxynonenal has reported to be a common factor for inducing cell death in both the brain and pancreas as a cause of Alzheimer’s disease and type 2 diabetes. Increased levels of hydroxynonenal were reported in patients with type 2 diabetes (Miwa et al., 2000; Lou et al., 2020) or in the brains of patients with MCI and Alzheimer’s disease (Barone et al., 2012; Scheff et al., 2016), as well as in the plasma and cerebrospinal fluid of Alzheimer patients (Selley et al., 2002). Miwa et al. (2000) suggested that the excessive hydroxynonenal impairs glucose-stimulated insulin secretion in isolated pancreatic β-cells, and contributed to the β-cell death in type 2 diabetes. In recent years, increased levels of hydroxynonenal were detected in serum, plasma, blood, urine, cells, and tissues of human patients with type 2 diabetes by different methods (Dator et al., 2019; Ito et al., 2019; Dham et al., 2021). Using the monkey experimental paradigm, the authors recently found that the long-term injections of the synthetic hydroxynonenal can cause not only neuronal degeneration but also Langerhans cell degeneration by oxidizing Hsp70.1 (Figure 5) (Boontem and Yamashima, 2021; Yamashima, 2021). In the brain, the vicious circle is established between the impaired insulin signaling system and the neurotoxic ingredients as amyloid *ß* and pTau. Intriguingly, all of these three (hydroxynonenal, amyloid β, and pTau) are capable of activating μ-calpain. To explain the close relation between Alzheimer’s disease and type 2 diabetes, the authors speculate that ‘calpain-mediated cleavage of oxidized Hsp70.1’ may occur in both the brain and pancreas (Yamashima et al., 2020). It is probable that the common causative factor of Alzheimer’s disease and type 2 diabetes is ‘hydroxynonenal’.

## Non-alcoholic steatohepatitis (NASH)

NASH is a progressive subtype of non-alcoholic fatty liver disease (NAFLD), being first defined by analogy to alcoholic hepatitis. However, this disease occurs in persons who consume little or no alcohol. NASH is characterized by the accumulation of fat in the liver (steatosis) along with inflammation and different degrees of scarring or fibrosis (Chalasani et al., 2017). The occurrence of NAFLD is associated with obesity, insulin resistance, and dyslipidemia, and its incidence is currently prevalent in the Western countries (Younossi et al., 2016). Since NASH-related liver cirrhosis and hepatocellular carcinoma are nowadays increasing, NASH is emerging as a world health problem (Estes et al., 2018). Multiple causative factors have been implicated in the pathophysiology of NAFLD. Although the underlying mechanism how NAFLD progresses to NASH is still not fully understood, accumulated evidence has suggested that oxidative stress is involved in this process (Seki et al., 2002; Rolo et al., 2012; Serviddio et al., 2013; Liu et al., 2015; Bellanti et al., 2017; Bekyarova et al., 2019). In the liver damage caused by a variety of hepatotoxic drugs and solvents, lipid peroxidation is considered a key factor for damaging hepatocytes, and the generation of reactive intermediates is a common event (Coleman et al., 2007). However, not only the mechanism underlying hepatocyte degeneration/death but also the role of toxic lipid peroxidation product ‘hydroxynonenal’ in NASH, long remained unclear.

Ensuing excessive ROS production enhances lipid peroxidation to elevate the concentration of hydroxynonenal, and cause hepatocyte damage and liver injury (Wang et al., 2015; Castro et al., 2017). Hydroxynonenal is generated mainly from linoleic acid, and is one of the most cytotoxic aldehydes for the liver (Mattson, 2009; Czerwińska et al., 2014; Castro et al., 2017). However, the molecular mechanism of hydoxynonenal-induced hepatocyte injury has not been elucidated. Chronic fructose consumption was found to cause fat accumulation in the liver (Bekyarova et al., 2017). Surplus of fatty acids in the fatty liver leads to mitochondrial production of excessive ROS which generate highly toxic hydroxynonenal. Significant increase in the serum hydroxynonenal levels has been demonstrated in the NASH patients, compared to those with simple steatosis (Videla et al., 2004).

**FIGURE 6:**
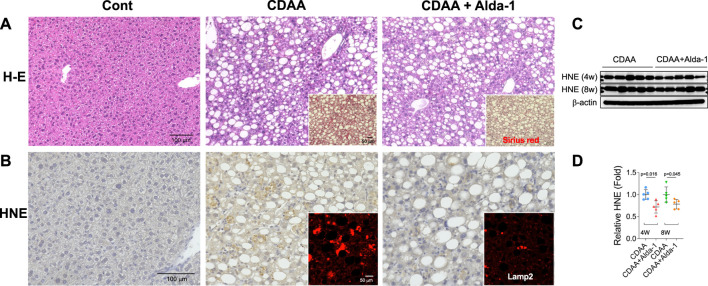
Alda-1 in CDAA mice suppresses liver fibrosis (Panel A and lysosomal disintegrity (Panel B). Panels **(A, B)** rectangles: CDAA mice show fibrosis on the Sirius red staining and lysosomal permeabilization on the Lamp-2 staining, while Alda-1 treatment (CDAA + Alda-1) disclosed decreased immunoreactivity of not only Sirius red and Lamp-2 but also HNE. Panel **(C)** Western blotting analyses of liver hydroxynonenal protein adducts in CDAA mice (CDAA) and CDAA mice with Alda-1 treatment (CDAA + Alda-1). Alda-1 treatment discloses decreased adducts. Panel **(C)** Each bands were quantified and shown as relative fold ratios. Adapted with permission from Seike et al. (2022).

**FIGURE 7:**
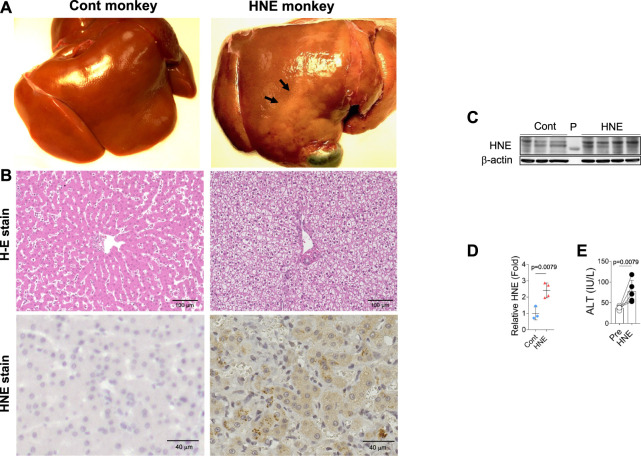
Hydroxynonenal (HNE) induces liver injury in the Japanese macaque monkeys. Panel **(A)** Macroscopic findings of livers of the control (Cont) and HNE-treated (HNE) monkeys. Black arrows show nodular discoloration. Panel **(B)** H-E staining and HNE immunostaining of liver tissue from the control group (Cont) and HNE-treated (HNE) group. HNE immunoreactivity was negligible in the control hepatocytes, but was distinct in the latter hepatocytes. Panel **(C)** Western blotting analyses of the liver HNE protein adducts in the control (Cont) and HNE-treated (HNE) group. P, protein marker. Panel **(D)** Bands of panel **C** were quantified and shown as relative fold ratios Panel **(E)** ALT showed a significant increase after hydroxynonenal injections (HNE), compared to the control (Pre). Adapted with permission from Seike et al. (2022).

**FIGURE 8:**
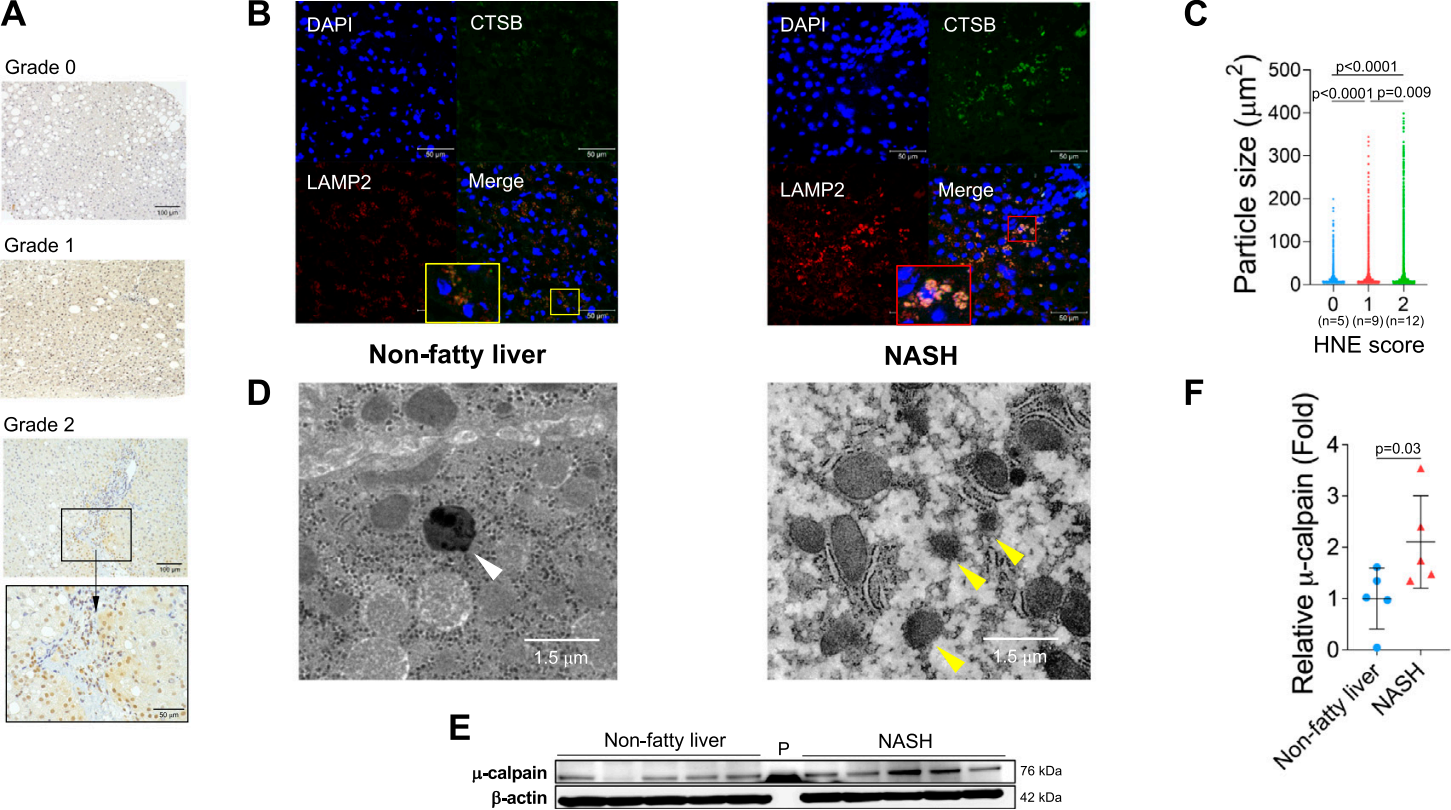
Hydroxynonenal (HNE) is involved in the progression of disease in human NASH. Panel **(A)** Semi-quantitative assessment of HNE immunoreactivity in the liver tissue of patients with non-fatty liver disease (NAFLD). The density of HNE immunoreactivity was scored into 3 grades: no staining (Grade 0), weak and uniform staining (Grade 1), and intense spots (rectangle) with uniform staining (Grade 2). Panel **(B)** Immunofluorescence staining of the liver tissue from patients with non-fatty liver and NASH shows that lysosomal membrane permeabilization/rupture was negligible in the former (yellow rectangle), but occurred remarkably in the latter (red rectangle). Blue, DAPI; green, cathepsin B (CTSB); red, Lamp-2. Panel **(C)** Relationship between the HNE staining score and double-stained granule sizes for Lamp-2 and cathepsin **(B)**. Panel **(D)** Electron microphotographs of the non-fatty liver and NASH liver. Lysosomes with distinct limiting membrane structures were observed in the non-fatty liver (white arrowhead). In contrast, lysosomes in the NASH liver showed disintegrity of the lysosomal membrane (yellow arrowheads). Panel **(E)** Western blotting analysis of μ-calpain in the liver tissues of non-fatty liver and NASH, shows an increased activation of μ-calpain in NASH. P, protein marker. Panel **(F)** Bands of panel E are quantified and shown as relative fold ratios. Adapted with permission from Seike et al. (2022).

From diverse experimental paradigms focusing hydroxynonenal-treated hepatocellular carcinoma cell lines, CDAA diet-fed NASH model mice (Figure 6), monkeys after the consecutive injections of synthetic hydroxynonenal (Figure 7), and human NASH patients (Figure 8), Seike et al. (2022) recently reported that hydroxynonenal can induce hepatocyte death due to the lysosomal membrane permeabilization/rupture. They demonstrated that hydroxynonenal is involved in the pathogenesis of NASH by activating μ-calpain *via* G-protein coupled receptor 120 (GPR120) and disrupt the lysosomal membrane with the resultant leakage of cathepsin enzymes causing hepatocyte death. Blockade of GPR120 or μ-calpain expression could suppress lysosomal membrane disintegrity and inhibit hepatocyte degeneration/death. Administration of Alda-1 (Chen et al., 2008; Perez-Miller et al., 2010), which activates ALDH2 to degrade hydroxynonenal, could reduce liver fibrosis as well as hydroxynonenal-induced lysosomal disintegrity and inflammation (Figure 6). Interestingly, in the monkeys after the long-term injections of the synthetic hydroxynonenal, the liver showed heterogenous discoloration which histologically comprised of nodular fatty degeneration with depositions of hydroxynonenal (Figures 7A,B). Furthermore, in the biopsied liver specimens from the NASH patients also, the degree of hydroxynonenal deposition in hepatocytes was more severe in cases with high scores of the lobular inflammation, ballooning and fibrosis, and was closely related to the extent of lysosomal rupture (Figures 8B,C) (Seike et al., 2022).

## Calpain-mediated cleavage of carbonylated Hsp70.1

**FIGURE 9:**
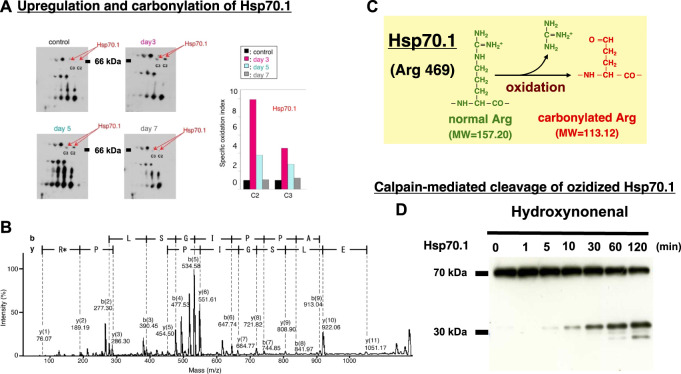
Upregulation, oxidation, and cleavage of Hsp70.1 after transient brain ischemia. Panel **(A)** Two-dimensional gel electrophoresis with immunoblot detection of carbonylated protein analysis (2D Oxyblot) of the postischemic hippocampal CA1 tissues after immunoprecipitation with anti-Hsp70.1 antibody, shows upregulation of carbonylated Hsp70.1 on the postischemic days 3 (pink) and 5 (blue), compared to the control (black). The specific oxidation index is significantly high on days 3 and 5. Panel **(B)** Matrix-assisted laser desorption ionization-time of flight/time of flight (MALDI-TOF/TOF) analysis of the upregulated spots with the Mascot search. Both the peptide sequence of the carbonylated peptide ion (459-FELSGIPPAPR*G-470) and the presence of y2 fragment ion atm/z 113.12, indicates that carbonylation occurred at Arg469 in Hsp70.1. R*: Carbonylated arginine Panel **(C)** In response to hydroxynonenal being generated by ROS, carbonylation occurred at the key site, Arg469 of Hsp70.1. A decrease of its molecular weight from 157.20 to 113.12 is compatible with the insult of carbonylation (Panels A,B,C: cited from Oikawa et al., 2009). Panel **(D)** *In-vitro* cleavage of Hsp70.1 by activated μ-calpain in brain tissues from the non-ischemic monkey. It is likely that hydroxynonenal-induced carbonylation facilitates calpain-mediated cleavage of the carbonylated Hsp70.1. Reprinted with permission from Sahara and Yamashim (2010); Yamashima et al. (2014).

In diverse experimental models, μ-calpain activation brought about necrotic cell death *via* the lysosomal membrane permeabilization/rupture and the resultant leakage of cathepsin enzymes. Similar lysosomal membrane disintegrity was confirmed to occur by the calpain-cathepsin cascade also in the neurodegeneration model of *Caenorhabditis elegans* (*C. elegans*)*.* In this model, loss of function of the proteases CLP-1 and TRA-3 (equivalent to calpains in *C. elegans*) as well as ASP-3 and ASP-4 (equivalent to cathepsins in *C. elegans*) was neuroprotective (Syntichaki et al., 2002). The ‘calpain-cathepsin hypothesis’ was originally formulated in 1998 (Yamashima et al., 1998), but the substrate protein of calpain at the lysosomal membrane was initially unknown. Ten years later, however, the proteomics analysis comparing the hippocampal tissues of monkeys before and after transient global brain ischemia, disclosed that the target molecule of activated μ-calpain is Hsp70.1. The postischemic hippocampus showed a remarkable upregulation of Hsp70.1 a few days after transient ischemia on the 2-D oxyblot analysis (Figure 9A). Furthermore, the proteomics analysis (Matrix-assisted laser desorption ionization-time of flight/time of flight analysis) showed a decrease of its molecular weight from 157.20 to 113.12, so the specific oxidative injury ‘carbonylation’ was identified at the Arg469 of Hsp70.1 due to the oxidative stress during the reperfusion phase (Figures 9B,C) (Oikawa et al., 2009; Yamashima and Oikawa, 2009). In addition, using brain tissues of monkeys, the calpain-mediated cleavage of the carbonylated Hsp70.1 was demonstrated to occur *in vitro* in parallel with hydroxynonenal-induced carbonylation (Figure 9D) (Yamashima et al., 2014; Liang et al., 2016). As calpain alone without hydroxynonenal-treatment (Figure 9D, time point ‘0’) showed no cleavage of non-oxidized Hsp70.1, hydroxynonenal-mediated carbonylation obviously facilitated calpain-mediated cleavage of Hsp70.1. Since Hsp70.1 cleavage was blocked by the specific calpain inhibitor N-acetyl-Leu-Leu-Nle-CHO (ALLN) dose-dependently, Hsp70.1, especially after the oxidative modification, was thought to be susceptible to cleavage by activated μ-calpain (Sahara and Yamashima, 2010; Yamashima, 2013; Yamashima et al., 2014).

**FIGURE 10:**
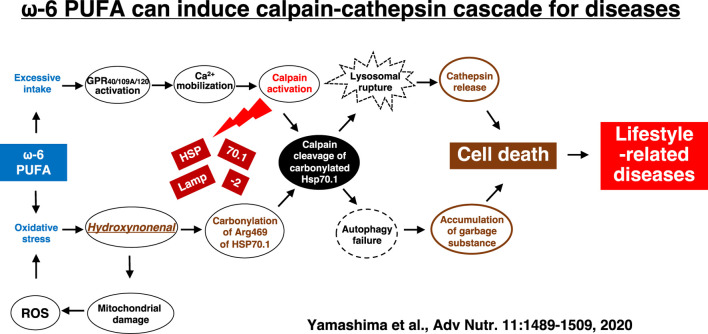
The calpain-cathepsin cascade explaining the molecular mechanism from ω-6 fatty acid-rich PUFA to cell death in lifestyle-related diseases. Diverse G protein-coupled receptors as GPR40/109A/120 in the brain/pancreas/liver are related to Ca^2+^ mobilization in response to fatty acids. Simultaneously, circumferential oxidative stress and/or deep frying may cause oxidation of ω-6 fatty acid with the resultant generation of hydroxynonenal. Hsp70.1 is a stress-induced protein or lysosomal stabilizer that confer cell protection against diverse stimuli, but its dysfunction caused by calpain-mediated cleavage of carbonylated Hsp70.1 induces diverse cell degeneration *via* lysosomal rupture and autophagy failure. It is probable that the same disorder may occur for the other lysosomal membrane proteins like Lamp-2. Adapted with permission from Yamashima et al. (2020).

Hydroxynonenal-mediated carbonylation of Hsp70.1 plays a supportive but crucial role for facilitating the calpain-mediated Hsp70.1 cleavage in the postischemic neurons (Sahara and Yamashima, 2010). As Hsp70.1 has dual functions as a chaperone protein and lysosomal membrane stabilizer, the Hsp70.1 disorder induce cell degeneration/death *via* the lysosomal membrane disintegrity with the resultant release of cathepsin enzymes. In addition, accumulation of garbage proteins occurs by the autophagy failure due to Hsp70.1 disorder (Adapted with permission from Figure 10). Presumably, the molecular mechanism of *ß* cell degeneration/death in type 2 diabetes can be explained also by the ‘calpain-cathepsin hypothesis’, because both calpain activation and extralysosomal leakage of cathepsin B were confirmed in the monkey pancreas after the consecutive injection of hydroxynonenal (Figure 5) (Boontem and Yamashima, 2021). However, there are still some limitations to explain the molecular mechanism of NASH by the calpain-mediated cleavage of the oxidized Hsp70.1, because in the damaged liver tissues at the advanced stage of disease (experiments), Seike et al. failed to confirm carbonylation and cleavage of Hsp70.1 disorder as demonstrated in the brain and pancreas (Seike et al., 2022). Unfortunately, they could not identify the substrate proteins which was oxidized (carbonylated) by hydroxynonenal and cleaved by activated μ-calpain at the lysosomal membrane of hepatocytes. It is conceivable that long term insults (exposure of 5 mg/week of hydroxynonenal for 24 weeks) was inappropriate to demonstrate calpain-mediated cleavage of the substrate proteins which presumably occurred in the earlier phase of exposure. If the timing of tissue sampling after the insult or during disease process is appropriate, calpain-mediated cleavage of the carbonylated Hsp70.1 would be demonstrated in diverse experimental models and human diseases.

The calpain-mediated cleavage of Hsp70.1 is physiologically indispensable for the turnover of cell proteins, but is detrimental for the cell survival when excessive. Activated μ-calpain was demonstrated in the previous studies to cleave not only Hsp70.1 (Oikawa et al., 2009; Yamashima and Oikawa, 2009; Zhu et al., 2014; Yamashima et al., 2020), but also Lamp-2 (Arnandis et al., 2012; Rodriguez and Torriglia, 2013; Gerónimo-Olvera et al., 2017), and v-ATPase subunit b2 (Arnandis et al., 2012) which are localized at the lysosomal membrane. Accordingly, in the NASH liver either of the latter two or all of the three lysosomal membrane proteins might be the substrates of activated μ-calpain especially after the carbonylation by hydroxynonenal. Future studies are needed to elucidate this issue. Overall, the lysosomal membrane contains more than 100 proteins, which comprised of anchoring proteins, transporters, receptors, and enzymes (Schröder et al., 2010). Recent studies have uncovered a range of lysosomal membrane proteins that can influence lysosomal cell death (Mrschtik and Ryan, 2015). The influence of ROS, calpain, and hydroxynonenal upon diverse lysosomal membrane proteins should be studied further to understand the detailed mechanism of lysosomal cell death which should be related to the occurrence of lifestyle-related diseases.

## Conclusion

1) The ‘calpain-cathepsin hypothesis‘ initially suggested implication of calpain and cathepsin for the ischemic neuronal death of monkeys. Now, it can expand the lysosomal theory about the pathogenesis of lifestyle-related diseases such as Alzheimer’s disease, type 2 diabetes, and NASH.
2) ROS may initiate a chain of responses that results in generation of hydroxynonenal with the long-term protein damage. High-fat diets or deep-fried foods cooked by ω-6 PUFA-rich vegetable oils, may induce the calpain-cathepsin cascade for the occurrence of cell degeneration/death in the brain, pancreas, liver, etc.
3) Garbage proteins like amyloid β and pTau accumulate as byproducts of the autophagy failure due to Hsp70.1 disorder. These garbages in turn would facilitate calpain activation to promote the vicious cycle of programmed cell death.

## Future perspectives

1) At present, it is difficult to clarify whether the main source of hydroxynonenal production is intracellular (e.g., generated at biomembranes by the circumferential and/or intrinsic oxidative stress) or extracellular (e.g., incorporated into the serum *via* high-fat diets and deep-fried foods etc.). This should be studied further.
2) The ‘calpain-cathepsin hypothesis’ can cover most of the mechanism of necrotic cell death. However, further studies are necessary to completely elucidate the mechanism and pattern of lysosomal cell death specific for each lifestyle-related disease.
3) Investigating the impact of oxidation of the lysosomal membrane proteins, especially focusing Hsp70.1, Lamp-2, v-ATPase subunit b2, etc., will help elucidate the mechanisms responsible for the cell death in lifestyle-related diseases.

## Abbreviations

ALDH2, Aldehyde dehydrogenase 2; ALDH2*2, Aldehyde dehydrogenase 2 inactivating mutation; ALLN, N-acetyl-Leu-Leu-Nle-CHO; BMP, bis(monoacylglycero)phosphate; *C. elegans*, *Caenorhabditis elegans*; CDAA-diet, Choline-deficient amino-acid defined-diet; CA1, *Cornu Ammonis* 1; GPR120, G protein-coupled receptor 120; HbA1c, Hemoglobin A1c; Hsp70.1, Heat-shock protein 70.1; DAPI, 4′,6-Diamidino-2-phenylindole; HNE, Hydroxynonenal; IRS-1, Insulin response substrate protein; Lamp-2, Lysosome-associated membrane protein-2; JNK, c-Jun N-terminal kinase; LIMP-2, Lysosomal integral membrane protein type-2; MALDI-TOF/TOF, Matrix-assisted laser desorption ionization-time of flight/time of flight analysis; MCI, Mild cognitive impairment; NAFLD, Non-alcoholic fatty liver disease; NASH, Non-alcoholic steatohepatitis; PUFA, Polyunsaturated fatty acids; pTau, Hyperphosphorylated Tau; zVAD-fmk, Z-Val-Ala-Asp-fmk; ROS, Reactive oxygen species.
